# A case of vestibular schwannoma with oral burning sensation: surgical complication or burning mouth syndrome?

**DOI:** 10.1186/s13030-021-00215-0

**Published:** 2021-08-23

**Authors:** Takayuki Suga, Trang T.H Tu, Junichiro Sakamoto, Akira Toyofuku

**Affiliations:** 1 Department of Psychosomatic Dentistry, Graduate School of Medical and Dental Sciences, Tokyo Medical and Dental University, 1-5-45 Yushima, Bunkyo-ku, 113-8510 Tokyo, Japan; 2 Department of Oral and Maxillofacial Radiology, Graduate School of Medical and Dental Sciences, Tokyo Medical and Dental University, Yushima 1-5-45, Bunkyo-ku, Tokyo, Japan

**Keywords:** Burning mouth syndrome, Acoustic neuroma, Vestibular Schwannoma, Tumour, Chronic pain

## Abstract

**Background:**

Despite improvements in surgical techniques, the removal of vestibular schwannoma is related to some complications. Recovery from surgical complications of vestibular schwannoma is often difficult and complications sometimes lead to permanent deficits. However, treatable trigeminal symptoms may be missed in atypical cases.

**Case presentation:**

A 46-year-old woman complained about burning sensation on her tongue and maxilla for four years before her first visit to our clinic. She visited the neurosurgery department in a university hospital because her facial pain and burning sensation of her tongue were suddenly aggravated. She was diagnosed with vestibular schwannoma and tumour resection was performed. However, her oral pain persisted after surgery. Two months before the initial visit to our clinic, the oral pain became more severe than ever before. When the patient visited a psychiatrist due to a panic attack, the psychiatrist diagnosed her as having somatic symptom disorder and depression and referred her to our clinic.

Based on the characteristics of the pain, she was diagnosed as burning mouth syndrome and treated for the same. Within 1.5 months, the pain and burning sensation of the tongue and maxilla almost completely remitted with low dose amitriptyline.

**Conclusions:**

Our case suggests that there are exceptional cases in which burning mouth syndrome and vestibular schwannoma occur simultaneously. Burning pain after vestibular schwannoma surgery cannot always be considered a complication of surgery.

## Background

Vestibular schwannoma (VS) or acoustic neuroma is a benign brain tumour that accounts for approximately 13 % of intracranial tumours and 90 % of tumours in the cerebellopontine angle [1, 2]. Despite improvements in surgical techniques, the removal of VS is associated with complications such as headaches, hearing loss, facial weakness, and other issues depending on the patient’s sex, age, and tumour size [3]. Wiegand et al. reported the surgical complications of VS and its treatment and suggested that the recovery from complications is often difficult and complications may sometimes lead to permanent deficits [4].

Burning Mouth Syndrome (BMS) presents as an uncomfortable sensation or oral burning pain in the mouth without clear clinical causes or pathophysiology. BMS mainly affects middle-aged and elderly women. About 10.7 % of complications of vestibular schwannoma surgery have been reported to affect the facial or tongue area, including numbness or pain, which is similar to the typical symptoms of BMS. However, to the best of our knowledge, there is no documented case of BMS either comorbid with vestibular schwannoma or that occurred due to surgical complications. Here, we report a case where VS and BMS coexist together.

## Case report

**Fig. 1:**
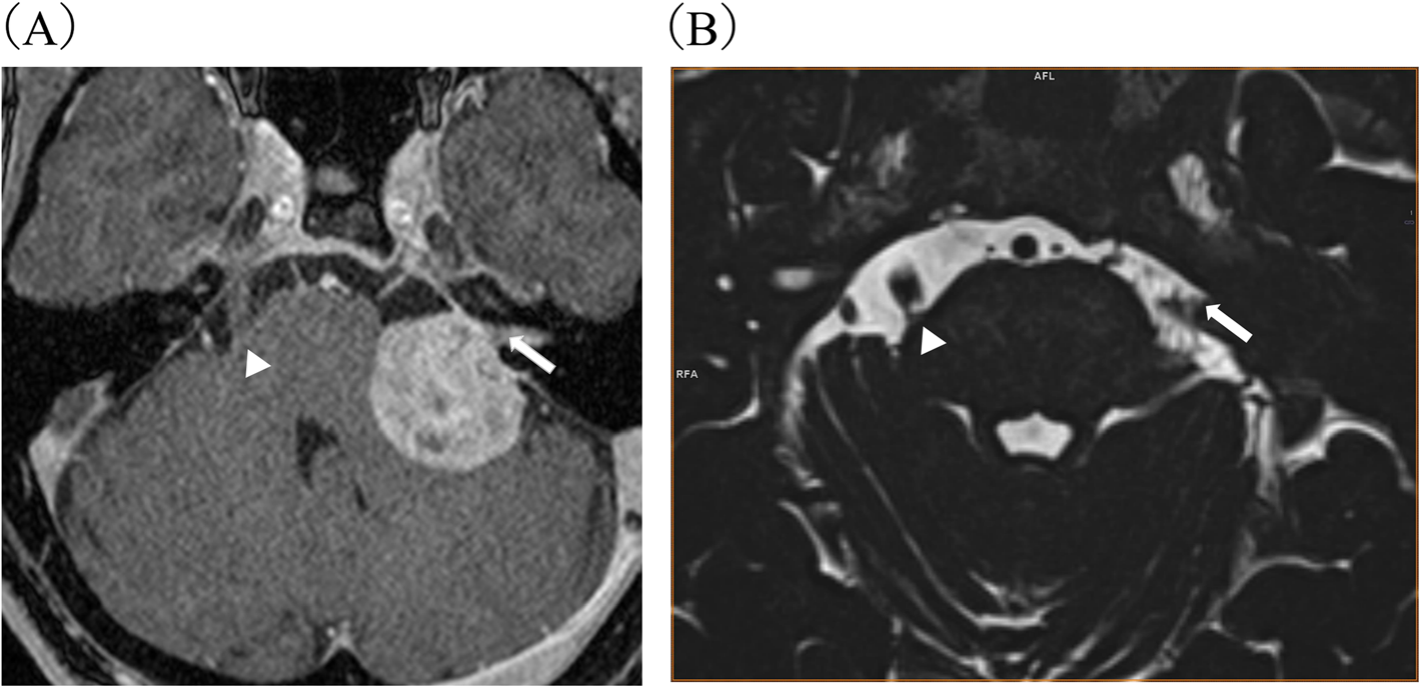
(**A**) Magnetic resonance imaging scans of VS (4 years before the patient’s first visit to our clinic) (well-defined mass involving the left cerebellopontine angle) (white arrow: vestibular schwannoma affecting the left trigeminal nerve, arrow head: right trigeminal nerve) (**B**) Magnetic resonance imaging scans from the postoperative period (white arrow: left trigeminal nerve, arrow head: right trigeminal nerve)

A 46-year-old woman reported with a complaint of burning pain at the back of her nostril, a purely burning sensation in her tongue and maxilla, and a spontaneous bitter taste. The symptoms were limited to the left side of her face. The pain did not include a shooting characteristic and paralysis was absent. However, it showed daily fluctuation, with relief in the morning and worsening at night. Four years before the patient’s first visit to our clinic, the facial pain and burning sensation of the tongue suddenly became aggravated without any recognizable triggering factor. She visited the neurosurgery department in a university hospital and VS was found localized in the left cerebellopontine angle (Fig. 1 (A)). The left trigeminal nerve was involved in the mass. Following a near total removal of the tumour, the facial pain, burning sensation of the tongue, and bitter taste improved, but soon recurred.

Over the four years before reporting to our hospital, facial pain, burning tongue and bitter taste often fluctuated, but they were bearable. A brain surgeon prescribed mecobalamin which was ineffective. Two months before the initial visit to our clinic, the pain became more severe than ever before. The patient started visiting a psychiatrist due to a panic attack, and quit her job as a nurse. The psychiatrist diagnosed her as having somatic symptom disorder and depression, prescribing sertraline 100 mg, zolpidem 5 mg, and lorazepam 1.0 mg and referring her to our clinic.

## Diagnosis and treatment course

The patient had no other significant medical history. At the first visit, no abnormal intraoral or extraoral findings were detected. The Saxon test’s result was 2.03 g/2min; which was in the normal range. Semmes-Weinstein monofilament testing on the cutaneous distribution of the second and third branch of the trigeminal nerve also revealed no sensory abnormality. There was no ulcer, redness or swelling of the oral mucosa. The visual analogue scale for pain, pain catastrophizing scale, and Zung’s self-rating depression scale scores were 48, 20, and 64, respectively. Eating food eased the symptoms. While taste perception was within the normal limit on the right side, filter-paper disk test revealed severe loss of taste on the left side of the tongue, where the patient could not sense sweet or bitter tastes and barely sensed salty or sour ones. The left trigeminal nerve was not depicted clearly on Magnetic resonance imaging (Fig. 1 (B)). The differential diagnosis was secondary trigeminal neuropathy after VS surgery. However, based on the absence of peripheral neuropathic symptoms like sensory loss, loss of muscle volume and the characteristics of the oral pain, such as its daily fluctuation, non-shock-like nature and attenuation with eating and sleeping, the symptoms seemed not to fit well with typical neuropathy pain. Thus, we made a diagnosis of BMS comorbid with atypical facial pain. Due to partial remission owing to sertraline, amitriptyline was initiated at 10 mg/day and increased to 30 mg with careful observation of its effectiveness and side effects. Within 1.5 months, the facial pain and burning sensation of the tongue and maxilla had almost remitted completely, whereas bitter taste showed moderate improvement. She stated that she could return to her job and even enjoy a family trip, which had previously been beyond her wildest dreams. In the subsequent 2 years, the patient has continued to take amitriptyline 30 mg/day without any side effects, and her facial pain and burning sensation of the tongue have disappeared completely.

## Discussion

About 8.6 % of patients experience non-audiofacial neurological complications such as neuropathy after VS surgery [5]. Pain in the trigeminal nerve area is known to be a possible complication of VS surgery [6].The cause-and-effect relationship between VS and BMS cannot be completely denied in this case because the onset was not clear due to the complicated history and because the symptoms were laterality to the left side.

Burning sensation and dysgeusia are common characteristics of VS and BMS. However, for differential diagnosis, the daily fluctuation is the biggest difference between burning sensation after VS and that of BMS [7]. Though the burning sensation of VS is usually trigeminal neuralgia-like that presents as paroxysmal rather than fluctuating [8], that of BMS has little pain in the morning with worsening throughout the day. It is eased with food intake and diminished while sleeping. In our case, her unilateral oral burning and dysgeusia could be explained by trigeminal nerve damage due to the VS [9]. However, considering her post-operative history, it would be more appropriate to regard the burning sensation mainly as a part of BMS.

According to the International Association of Pain and International Headache Society, the criteria for BMS diagnosis excludes organic abnormalities, such as brain tumours [10]. If clinicians follow these criteria, the symptoms of patients like the present case would be left untreated or regarded as complications after neurosurgery. Our case suggests there are exceptional cases in which BMS and VS can occur simultaneously. The symptoms of VS like oral burning and dysgeusia sometimes mimic those of BMS [7]. Hence, it is essential for pain clinicians to investigate thoroughly and consider every possibility as opposed to merely depending on the standard diagnostic criteria. Careful differential diagnosis is required for cases presenting BMS like symptoms. We will need to further investigate many more such cases of BMS comorbid with VS to develop a clear understanding of its unique nature.

## Data Availability

The dataset supporting the conclusions of this article is available in from the Department of Psychosomatic Dentistry, Graduate School of Tokyo Medical and Dental University.

## Abbreviations

BMS: Burning mouth syndrome
VS: Vestibular schwannoma
